# Supplementary material for: Test-Retest Reliability and Walk Score® Neighbourhood Walkability Comparison of an Online Perceived Neighbourhood-Specific Adaptation of the International Physical Activity Questionnaire (IPAQ)
Source: Int J Environ Res Public Health. 2019 May 30;16(11):1917. doi: 10.3390/ijerph16111917 (PMC6603616; doi:10.3390/ijerph16111917)
Supplement: Supplementary file 1 [file ijerph-16-01917-s001.pdf]

Participant ID:

Pathways to Health

The Pathways to Health Study: Neighbourhood Physical Activity Measurement Tool

Thank you for agreeing to complete this survey. This questionnaire collects important information about your physical activity, about your neighbourhood, and about you. We are interested in finding out about the kinds of physical activities that people do as part of their everyday lives.

All information you provide is strictly confidential. To make our study useful we need as many questions answered as possible; all questions if possible. We value your opinions. This questionnaire should take approximately 20 minutes to complete. Please read all questions carefully.

Instructions for maximum accuracy

Mark choice boxes with a cross like this: [X]

Mark response boxes with a value, for example: _ _ 9 9

If you make a mistake, cross out your incorrect choice like this: [~~X~~]

Use a pencil or ballpoint pen.

During the **last 7 days**, was there anything that altered your normal levels of physical activity (i.e. injury, travel, sickness, etc.)?

Yes ☐ No ☐

QUESTIONS ABOUT YOUR PHYSICAL ACTIVITY

The following questions will ask you about the time you spent being physically active in the **last 7 days**. Answer each question even if you do not consider yourself an active person. Think about the activities you do to get from place to place, and in your spare time for recreation, exercise or sport. Think about whether your activities were carried out **inside** your residential neighbourhood.

PART 1: TRANSPORTATION PHYSICAL ACTIVITY

These questions are about how you traveled from place to place, including to places like work, stores, movies, and so on.

1. During the **last 7 days**, on how many days did you **travel in a motor vehicle** like a train, bus, car?

|__|__|__|__| **days per week** *(if '0' skip to question 3)*

2. How much time did you usually spend on one of those days traveling in a train, bus, car, or other kind of motor vehicle?

|__|__|__|__| **hours per day**

|__|__|__|__| **minutes per day**

Now think only about the **bicycling** and **walking** you might have done to travel to and from work, to do errands, or to go from place to place **inside** your neighbourhood.

3. During the **last 7 days**, on how many days did you **bicycle** to go from place to place **inside** your neighbourhood?

|__|__|__|__| **days per week** *(if '0' skip to question 5)*

4. How much time did you usually spend on one of those days to bicycle from place to place **inside** your neighbourhood?

|__|__|__|__| **hours per day**

|__|__|__|__| **minutes per day**

5. During the **last 7 days**, on how many days did you **walk** to go from place to place **inside** your neighbourhood?

|__|__|__|__| **days per week** *(if '0' skip to question 7)*

6. How much time did you usually spend on <u>one of those days</u> walking from place to place **inside** your neighbourhood?

|__|__|__|__| **hours per day**

|__|__|__|__| **minutes per day**

PART 2: RECREATION, SPORT, AND LEISURE-TIME PHYSICAL ACTIVITY

This section is about all the physical activities that you did in the **last 7 days** solely for recreation, sport, exercise or leisure. Please do not include any activities you have already mentioned.

7. Not counting any walking you have already mentioned, during the **last 7 days**, on how many days did you **walk** for **recreation, leisure, or exercise inside** your neighbourhood?

|__|__|__|__| **days per week** *(if '0' skip to question 9)*

8. How much time did you usually spend on <u>one of those days</u> walking for recreation, leisure, or exercise **inside** your neighbourhood?

|__|__|__|__| **hours per day**

|__|__|__|__| **minutes per day**

Now consider all the **moderate** and **vigorous** activities that you did in the **last 7 days**.

Moderate activities include activities that take moderate physical effort and make you breathe somewhat harder than normal (you could still talk to someone during the activity).
Vigorous activities include activities that require hard physical effort and make you breathe much harder than normal (it would be very difficult for you to talk to someone during the activity).

9. During the **last 7 days**, on how many days did you do **vigorous** physical activities like aerobics, running, fast bicycling, or fast swimming in your **leisure** time **inside** your neighbourhood?

|__|__|__|__| **days per week** *(if '0' skip to question 11)*

10. How much time did you usually spend on one of those days doing **vigorous** physical activities in your leisure time **inside** your neighbourhood?

|___|___|___|___| **hours per day**

|___|___|___|___| **minutes per day**

11. During the **last 7 days**, on how many days did you do **moderate** physical activities like bicycling at a regular pace, swimming at a regular pace, and doubles tennis in your **leisure** time **inside** your neighbourhood?

|___|___|___|___| **days per week** *(if '0' skip to question 13)*

12. How much time did you usually spend on one of those days doing **moderate** physical activities in your leisure time **inside** your neighbourhood?

|___|___|___|___| **hours per day**

|___|___|___|___| **minutes per day**

PART 3: TIME SPENT SITTING

These questions are about the time you spend sitting while at home. Do not include sitting time outside of your home or in a motor vehicle. Include any time spent sitting at a desk, using a computer, playing video games, reading or sitting or lying down to watch television at home.

13. During the **last 7 days**, how much time did you usually spend **sitting** on a **weekday**?

|___|___|___|___| **hours per day**

|___|___|___|___| **minutes per day**

14. During the **last 7 days**, how much time did you usually spend **sitting** on a **weekend day**?

|___|___|___|___| **hours per day**

|___|___|___|___| **minutes per day**

Thank you for completing this survey
